# 

**Published:** 1917-11

**Authors:** 


### TABLE OF CONTENTS

War Work of the American Medical Women ____________221

Nasal Obstruction a Factor in the Production of Disease __227

Control of Venereal Disease in Australia ________________232

Crawford Williamson Long and Ether _________________233

Medical Items ______________________________________252

